# HealthyBlock: Blockchain-Based IT Architecture for Electronic Medical Records Resilient to Connectivity Failures

**DOI:** 10.3390/ijerph17197132

**Published:** 2020-09-29

**Authors:** Omar Gutiérrez, Giordy Romero, Luis Pérez, Augusto Salazar, Marina Charris, Pedro Wightman

**Affiliations:** 1 Department of Systems Engineering, University of the North, Barranquilla 081007, Colombia; giordyr@uninorte.edu.co (G.R.); lalinares@uninorte.edu.co (L.P.); augustosalazar@uninorte.edu.co (A.S.); 2 CNCA Research Group, The National Training Service (Servicio Nacional de Aprendizaje—SENA), Barranquilla 080005, Colombia; 3 Department of Research in Telematics and Telecommunications, LS Information Technology Consulting (LS Asesorías TI), Barranquilla 080020, Colombia; marinacharris@lsasesoriasti.com

**Keywords:** blockchain, electronic medical records, EMR, resilience, information technology architecture, data integrity

## Abstract

The current information systems for the registration and control of electronic medical records (EMR) present a series of problems in terms of the fragmentation, security, and privacy of medical information, since each health institution, laboratory, doctor, etc. has its own database and manages its own information, without the intervention of patients. This situation does not favor effective treatment and prevention of diseases for the population, due to potential information loss, misinformation, or data leaks related to a patient, which in turn may imply a direct risk for the individual and high public health costs for governments. One of the proposed solutions to this problem has been the creation of electronic medical record (EMR) systems using blockchain networks; however, most of them do not take into account the occurrence of connectivity failures, such as those found in various developing countries, which can lead to failures in the integrity of the system data. To address these problems, HealthyBlock is presented in this paper as an architecture based on blockchain networks, which proposes a unified electronic medical record system that considers different clinical providers, with resilience in data integrity during connectivity failure and with usability, security, and privacy characteristics. On the basis of the HealthyBlock architecture, a prototype was implemented for the care of patients in a network of hospitals. The results of the evaluation showed high efficiency in keeping the EMRs of patients unified, updated, and secure, regardless of the network clinical provider they consult.

## 1. Introduction

Health can be defined as a state of complete physical, mental, and social wellbeing, and not just the absence of disease or illness [1]. To achieve the above, each person should keep an appropriate record of the medical controls and treatments they have received throughout their lives; however, this does not happen. Most medical information systems are not interconnected, and each health center, laboratory, specialist doctor, etc. has its own private database, with restricted access for patients. This situation leaves the patients with complete responsibility to remember all their prior and current conditions and treatments. This becomes a problem when, during each visit to a health system actor, it is necessary to rerecord the patient’s medical history, risking omissions with or without intention. This fragmented view also prevents a historical analysis from being used to support new diagnostic processes.

In this context, and with the advancement of eHealth technology [2], electronic medical records (EMRs) have emerged as the first part of a solution to offer the integration of a person’s clinical information [3]. However, it is not enough since each health entity records the clinical information of patients individually, disconnected from other entities. In addition, we must add the potential inconsistency of the information among medical entities, as well as loss of data and the lack of control of patients over who can see their clinical history [4]. That is, patients, despite being the main actor protected by laws in many countries [5,6], have limited access to and little control over their health information [7]. Putting all these problems together results in low effectiveness of the health system for the treatment and prevention of diseases that affect the general population, which in turn generates high costs in public health for governments, especially those in developing countries [8].

There are different proposals in the literature for electronic medical records (EMRs) [9,10,11]; however, this is a topic still open to research since the integration of these requires not only a unified data system, but the definition of policies and strategies that address current needs such as access to data by the patient (whereby the patient can see all their medical history in one place), the user’s participation in decisions regarding their information (access notifications and the possibility of defining restrictions to third parties), security and traceability of data (avoiding modifications to data), accessibility, and consistency.

A technology beginning to be used as a tool to solve some of these issues is blockchain. In 2009, this concept was unveiled by Satoshi Nakamoto [12], and it was initially used to store transactions originated by movements or changes in value of the Bitcoin cryptocurrency; however, the proposed structure can be used to exchange any set of values in a distributed and secure way. In general, a blockchain is a distributed system of nodes, with an immutable data structure, which allows the transaction log to be kept in a secure manner, among many actors [13]. Blockchain technology has been used not only in the financial field, but in various applications such as automatic driving of cars and vehicle networks [14], educational records [15], identity management [16], chains of supply [17], Internet of things (IoT) [18], and in the health area [19,20,21]. In the latter field, due to its characteristics, blockchain can support the development of EMR solutions that comply with the requirements defined above for these systems.

The advantages of blockchain in EMR were presented in different studies [22,23,24,25,26,27,28], in relation to the fragmentation, privacy, and scalability of data. However, the solutions proposed in the literature assume stable and permanent connectivity environments, which may not be the case for developing countries [29], where, for reasons ranging from technical to economic, communication networks may not be stable over a period of time, which generates connection failures that can be intermittent or for long periods of time. Taking the above into account, and after analyzing the workflows of the systems found in the literature, it can be seen that they do not contemplate solutions to connection failures; thus, when facing a disconnection from the blockchain network, these systems in some cases are unable to function properly. This situation could lead to the non-provision of services to patients or clinical actors and, in other cases, could lead to the loss of integrity between the data stored in the local databases of the providers (hospitals, clinics, laboratories, external doctors, etc.) and those replicated in the blockchain.

In the present work, HealthyBlock is proposed as an architecture based on blockchain networks that allows the development of electronic medical record systems shared between different clinical providers, with resilience in data integrity in the event of connectivity failures and with usability, security, and privacy characteristics. Likewise, the application of the proposed architecture is presented in the development of an electronic medical records (EMR) system for the care of patients in a network of hospitals and the evaluation of this prototype.

The conceptual development of the proposed architecture was done through the use of the Archimate visual modeling language, which allows describing, analyzing, and communicating business architectures [30]. For the prototype carried out with the HealthyBlock architecture, a permissioned blockchain network was created using Ethereum [31], with smart contracts for the management of users and medical records of patients. For the development of the front-end development, ReactJS [32] was used, and PostgreSQL [33] was used as the database system for local data storage. For the evaluation of the prototype performance, parameters such as system latency, performance, and resource utilization were measured.

The rest of this document is organized as follows: in Section 2, preliminary theoretical concepts and related work to the development of blockchain architectures for health are presented. Section 3 presents the proposed HealthyBlock architecture. In Section 4, the development of a prototype based on the HealthyBlock architecture is presented. Section 5 addresses the environment and the tests carried out on the prototype developed using the HealthyBlock architecture. Finally, Section 6 and Section 7 are devoted to the discussion on the proposed architecture, conclusions, and future work.

## 2. Literature Review

### 2.1. Background

#### 2.1.1. Electronic Medical Records (EMRs)

There are various concepts associated with EMR such as electronic health record (EHR), personal health record (PHR), electronic patient record (EPR), digital medical record (DMR), computer-based patient record (CPR), patient-carried record (PCR), and computerized medical record (CMR). Of all the previous concepts, the broadest is that of EHR, which is defined in the ISO/TR 20514: 2005 Health Informatics, Electronic Health Record [3] standard. This document defines EHR as a repository of information about a person’s health status in a format that can be processed by a computer, which is stored and transmitted in a secure and accessible way by multiple authorized users, having a standardized format that is independent of the electronic medical records system used and whose purpose is that of accompanying the continuity of care efficiently and facilitating quality integrated healthcare. For all purposes in this article, EMR is taken as equivalent to EHR.

#### 2.1.2. International Standards for EMRs

There are a series of international standards that allow regulating the aspects of content, structure, representation, distribution, security, confidentiality, authentication, and exchange, among other aspects, of electronic medical records; the most recognized are HL7 (Health Level Seven International) [34], ISO 18308: 2011 [35], and HIPAA (Health Insurance Portability and Accountability Act of 1996) [36].

#### 2.1.3. Blockchain

A blockchain is a distributed, encrypted, and shared database among a group of participants called nodes, which works like a ledger, keeping all network records or information in blocks [37]. In the blockchain, the information stored in all the nodes is the same and cannot be eliminated, which serves as a strategy for the protection and validation of the information. Likewise, the information is transmitted to the entire network, and the members who receive it must verify the integrity of the information, in order to be saved. The most common way of performing this task is called mining, which is the generation of new blocks in the network, which contain the new transactions and the inclusion of validation information that is verified by the rest of the nodes. To choose which of the different blocks generated by the miners is the correct one, a consensus algorithm is used, the concept of which is described in Section 2.1.5. There are three types of blockchain:

- Public or permissionless blockchain: public blockchain networks are those to which anyone has access [38].
- Private or permissioned blockchain: private blockchain networks are those where control is reduced to a single entity, responsible for maintaining the chain, giving permissions to the users that want to participate, proposing transactions, and accepting the blocks. A mining process does not exist [38].
- Hybrid or consortium blockchain: hybrid blockchains are not open to public participation, but a certain number of organizations, entities, or companies are responsible for managing the network as a whole and keeping synchronized copies of the registry [38].

#### 2.1.4. Smart Contract

Smart contracts are programs that govern transactions on the basis of a series of previously programmed rules [39]. This program is stored on the blockchain and runs automatically with each transaction. Smart contracts can have many contractual clauses that could be made partially or totally self-executing, or both. To use a smart contract in the blockchain network, the conditions are first programmed, then the parties involved are signed, and finally it is deployed on a blockchain such that it cannot be modified while being used by the blockchain [40].

#### 2.1.5. Consensus Algorithms

A consensus algorithm is a mechanism to determine whether the addition of a new block to the chain is valid or not [41]. The main consensus algorithms are proof of work (PoW) [42], proof of authority [43,44], proof of stake (PoS) [45], and practical byzantine fault tolerance (PBFT) [46], among others. In the present work, proof of authority (PoA) was used. In this algorithm, previously approved authority nodes called seals or validators are used that certify the transactions and blocks in the network [43]. The main characteristics of this algorithm are (1) minimal use of processing, since there is no need to solve complex operations to reach a consensus on which blocks to add to the network, (2) lower energy consumption, since the validators do not compete with each other to validate the blocks, (3) high performance, since its computational complexity is much lower, and (4) security, since the nodes in the blockchain only receive new blocks from the previously approved validator nodes.

#### 2.1.6. Connectivity Failure

In the present work, a connectivity failure is defined as an interruption between one of the nodes of the blockchain and the remaining nodes of the blockchain network. In the node that suffers the interruption, a copy of the blockchain is locally stored, which can still be accessed; however, information quickly becomes out of date compared to the copy of the blockchain of the nodes that are still connected to the network. This represents a problem because the disconnected node cannot process any new transactions into blocks (i.e., mine). because the new blocks containing this information will most likely become invalidated by the rest of the nodes in the network once the node reconnects; this means that all patient information will not be synchronized correctly with the blockchain, thereby creating a problem with the integrity of the data.

### 2.2. Related Work

This section discusses some existing proposals for blockchain-based architectures for EMRs. MedRec [22], developed by Massachusetts Institute of Technology (MIT) researchers, is a decentralized record management system, whose architecture offers the possibility of managing permits and data exchange between different healthcare systems, but keeping the clinical data of patients in the local electronic systems of hospitals and clinics. MedRec is supported by an Ethereum blockchain [31]. MedRec manages authentication, confidentiality, liability, and data exchange through the use of three smart contracts. In the first of these contracts called registrar (RC), the data of the identities of people who have some function within the clinical ecosystem are stored, as well as public keys. In the second contract called patient relationship provider (PPR), the relationships between the patient and the provider are established; this is issued when one node stores or manages data for another node, and it allows patients to select which part of their medical records they wish to share. The third contract called summary (SC) allows patients to access their medical history, described by its authors as a “breadcrumb trail” for participants in the system to locate their medical history, since it has a list of references to patient relationship provider (PPR) contracts, representing all past and current commitments of participants to other nodes in the system.

In [23], Medchain was proposed, which is a healthcare data exchange system that uses two separate decentralized networks: a blockchain network where the fingerprint of data, session, and operation is stored (data that are immutable) and a second peer-to-peer (P2P) storage network, where descriptions of the data and the session are stored, which are mutable and, therefore, can be updated. To achieve security and preservation of privacy, Medchain uses a session-based data exchange.

Patientory [24] is a patient-centered system supported by blockchain technology that enables the connection of centralized and isolated electronic medical records (EMRs) for the storage of medical records using distributed blockchain computing. Patientory implements HIPAA in relation to security and privacy; for this, it encrypts the information of the medical records of the patients in each of the medical service providers, whether they are hospitals or insurance companies. The architecture of Patientory works on a permissioned blockchain, in which each node is authorized to interact only with other nodes on the same network, with the HIPAA storage system, and with the RPC (remote procedure call) server; the latter is the connection interface between Patientory’s permissioned blockchain and the public internet network. In the proposed architecture, it also contains the key authoring entity, in charge of generating the private/public keys that will be used in the blockchain and the HIPAA database which stores the data that constitute private health information.

In [25], Dubovitskaya et al. proposed an architecture to manage and share electronic medical records of cancer patients using a blockchain network. This architecture is formed by a membership service whose function is to register users with different roles such as patient or doctor; in the latter case, the system verifies if the doctor is registered with The National Practitioner Data Bank, to check whether they are indeed a duly licensed physician. Likewise, the membership service is in charge of generating the keys used by the patient to sign their medical history and the encryption keys to guarantee the confidentiality of the medical records. Patient data are stored both locally in the database of the hospital where the patient is being treated and in a cloud-based platform, so that they are available to other members of the network. In the case of data storage in the cloud, these are stored encrypted with the patient key generated by the membership system. This framework also implements an application programming interface (API) for users, in charge of receiving the actions from the users and taking them to the nodes where they are organized by a leading node, which organizes them in blocks and initiates the consensus protocol. These transactions are replicated in all the nodes according to the logic programmed in each one of them.

The Ancile platform [26] is a system for the interoperability of electronic medical health records implemented on Ethereum, which also preserves the privacy of patient information. In Ancile, there are multiple parts that can interact through a private blockchain. Medical records are stored on the blockchain using hashes.

In MeDShare [27], the authors propose an architecture for the storage, privacy control, and exchange of medical data between providers in an environment without trust. MeDShare uses the blockchain network to save the history of the operations carried out on the data by the participants and smart contracts to enforce access control and, in case of misuse, revoke the access permissions granted.

There are other related papers [47,48,49,50], which proposed blockchain-based EMR architectures, that addressed different approaches to ensuring privacy in electronic medical record systems. Due to the above and given that privacy for electronic medical records is regulated by government laws [6,36], a review of the literature on this topic was carried out. In this review, it was found that a very important aspect is the type of access control to be used for the confidentiality of clinical information, mainly highlighting the role-based access control (RBAC) access control [51,52,53,54]. In this type of control, each user who accesses the system is assigned a role, which, in turn, has a defined series of permissions and restrictions. For the creation of this type of control, it is proposed that the institutions, hospitals, or some committee define them previously, while other studies suggest that it is the patient who may include refinements or restrictions on access permits to their clinical history, thereby customizing them according to their needs. Another important aspect of privacy is the way in which data are stored; for this purpose, [10] proposed a cloud storage scheme in encrypted form of electronic medical records, which contain personal information, diagnoses, medications, allergies, etc., along with attribute-based access policies to stored data. Likewise, the proposed schemes have re-encryption systems that allow authorized users, friends, family, doctors, and insurance providers, among others, to decrypt the requested EMR files. In [55], the authors proposed, for the secure storage of health data, a protocol using a symmetric encryption scheme that allows the secure storage of encrypted versions of a user’s medical data.

After analyzing the architectures found in the literature, some generalities can be identified:

- They are implemented to unite a set of geographically dispersed clinical providers.
- In some cases, these systems communicate with the existing EMR systems in clinical providers, especially with their local databases, and, in other cases, the proposed architecture completely replaces them.
- In most of the designs, blockchain is used as a repository of cryptographic hashes that act as pointers to indicate the place where medical records are stored in local databases; in other cases, EMRs are stored entirely on the blockchain.
- All systems propose strategies to guarantee the security and privacy of medical records, which is understandable given the sensitivity of clinical information.
- Generally, private blockchains are used, but some proposals also use public blockchains.
- Different consensus algorithms are used to mine or store transactions on the blockchain, the most widely used being proof of work (PoW).
- In each clinical provider, there is a blockchain node, which has a copy of the chain and, in order to improve efficiency, this node is a blockchain miner.

As can be seen, the systems found in the literature address different concerns about the creation of electronic medical record systems supported by blockchain technology; however, none of these contemplate the operation of the system when a blockchain node, located in a clinical provider, is disconnected from the blockchain network. This is critical because, during the time it remains disconnected, new transactions are generated at the node, which must be referenced or stored in the blockchain. The studies exposed in the literature neither contemplate this case nor indicate how their systems work under these conditions; however, from the analysis of the workflows shown in their publications and on the basis of the normal operation of blockchain networks, it can be inferred that the proposed systems, having no way of detecting that the node was disconnected from the blockchain, will continue to send transactions to it, in which case, there are two potentially critical problems:

- The first scenario occurs if the blockchain node that was disconnected is a mining node. In this case, the node acts as a fork of a single node of the blockchain; therefore, when receiving transactions, it proceeds to mine them on the local copy of the blockchain. Once this node reconnects with the blockchain network, the copy of the chain that it contains and that was used to store the transactions will not be valid for the rest of the blockchain network. This occurs because the remaining nodes of the chain act as the majority, and they have a different version of the blockchain than the one at the node; thus, the blockchain will proceed to synchronize and send the valid copy of the blockchain, overwriting the previous one. This will result in the loss of the last transactions created in the node of the clinical provider, which will lead to a failure in the integrity of the data between the blockchain and the node’s local database.
- The second problem occurs when the node that loses connectivity is a non-mining node. According to the operation of blockchains in general, the transactions received by a node, during the time it is not connected to the blockchain network, are stored by it and, once the node reconnects, it will proceed to send the transactions for mining. When contrasting this general operation of the blockchain with the analysis of the workflows of the EMR systems found in the literature, it can be observed that many of them need to receive confirmation from the blockchain nodes related to the mining of the transactions; however, as the node is disconnected, this confirmation cannot be generated and, therefore, the workflows will be interrupted.

Therefore, the systems found in the literature may not function properly.

Failures related to connection to the node of the blockchain network and their impact on the integrity of the system data are the main problems addressed in this work, whereby the proposed architecture takes into account the fragmentation, usability, security, and privacy of the systems of electronic medical records, using blockchain networks.

## 3. Proposed Architecture

### 3.1. Preliminaries

In this section, some preliminary concepts used for the development of the proposed architecture are presented.

#### 3.1.1. Software Components

**Figure 1:**
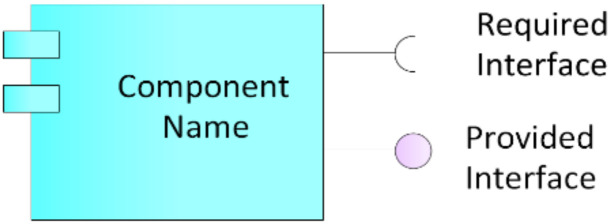
Representation of a component.

A component is a unit of a system that can be developed independently and, together with other components, gives functionality to a system through its interfaces [56]. Figure 1 shows the graphic representation of a component.

#### 3.1.2. Information Technology (IT) Architecture

The IT architecture is a view of the system that includes the main software components, as well as their behavior, interaction, and coordination to achieve the objective of the system [57]. There are different types of architectures, one of the most used being layered architecture, in which the system is organized in layers in such a way that the software components of each layer communicate with the software components of other layers through known interfaces [58], and each lower level provides its services only at a higher level if the design involves strict layers or at different levels if the design is layered.

#### 3.1.3. Archimate Modeling Language

Archimate is a visual language with a default iconography to describe, analyze, and communicate many concerns of enterprise architectures as they change over time [30].

#### 3.1.4. CRU (Create, Read, Update) Operations

This acronym is used throughout this paper to indicate the three operations that can be performed on digital data management in environments with blockchain networks: create, read, and update.

### 3.2. Architecture Overview

**Figure 2:**
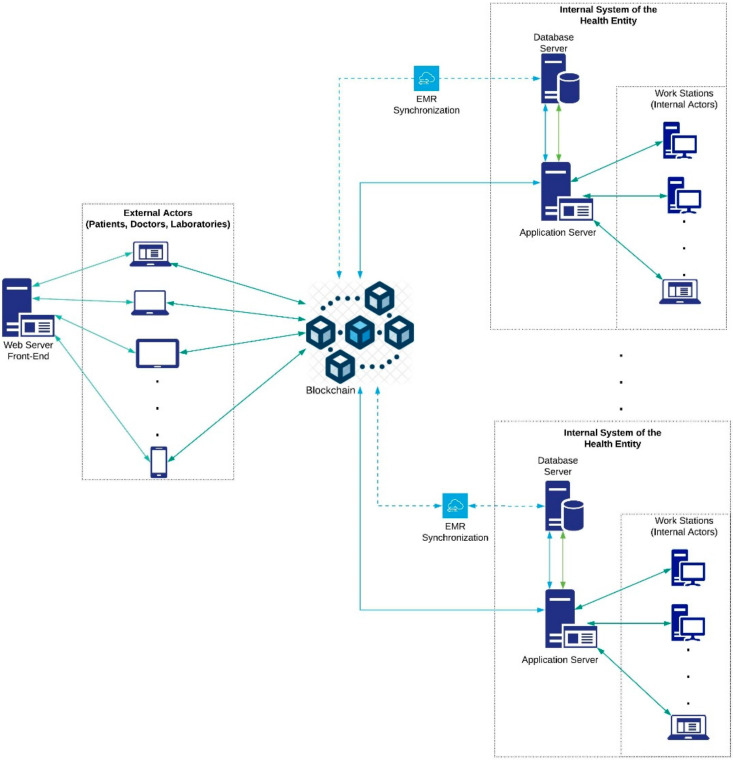
Architecture overview.

Figure 2 presents a general usage scenario for the proposed HealthyBlock architecture. In this graphic representation, the essential elements of implementation of the architecture can be seen, such as medical blockchain, responsible for storing the EMR, and health entities, which have database servers and applications that synchronize with the blockchain for the local storage of the EMRs. As it can be seen, the internal actors of these health entities such as doctors, nurses, and other authorized personnel communicate with the system through the internal application server. Likewise, the synchronizer component is in charge of keeping the medical records of the local databases of health entities unified with those of the blockchain, guaranteeing data integrity and resilience in the case of connectivity failures. Finally, there are external actors, such as patients, laboratories, and external doctors who communicate with the blockchain through an external web server. HealthyBlock is based on the following premises:

- The owner of the electronic medical records is the patient.
- The internal and external system actors are enrolled in the system by an administrative authority, which is designated by the participating entities of the system and is in charge of verifying the suitability of the enrollment of the actors who request it.
- When a patient enters the system, a family doctor is assigned. This doctor becomes a custodian and has full access to that patient’s electronic medical records.
- A patient may have an additional custodian of their EMRs, especially in cases when the patient is a minor or for emergencies.
- A patient’s medical records have an access control policy that can only be changed by the patient or the data custodians. This policy is recorded in the blockchain, as are the changes made to it and the author of the changes. In this way, the traceability of who had access to the data is ensured.

**Figure 3:**
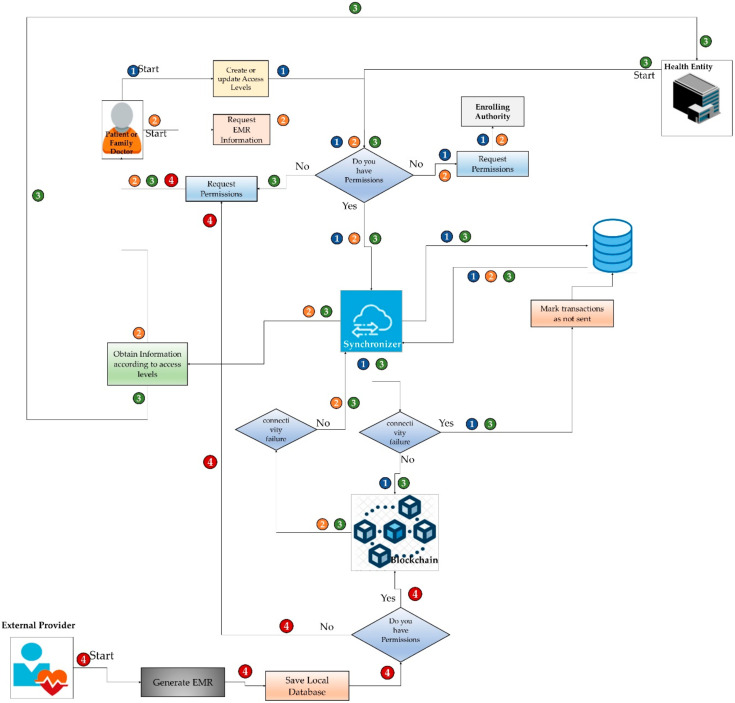
Interaction cases of the different actors.

The cases of interaction of the actors, both internal and external, with the HealthyBlock architecture are shown in Figure 3; a description of these cases is given below.

- Case 1. Patient or family doctor creates or updates access levels. This case starts when a patient or family doctor receives a request for access or wishes to give access to some actor in the system of their clinical data or those of their patient. To do this, the user enters the system, which asks for access data. The system verifies, according to the credentials, if the user has permission for the action to be performed; if so, it grants permission, and the patient, custodian or family doctor proceeds to make changes in the access levels, which are stored in the local database. Then, the synchronizer, if there is a connection to the network, proceeds to send the transactions to the blockchain; otherwise, it marks them as not sent before proceeding to send them as soon as there is a connection to the blockchain. In case of not having the required permissions, the system sends a request for authorization to the managing authority.
- Case 2. Patient or family doctor consults EMR. This case starts when the electronic medical records of a patient are going to be consulted by the patient herself or by its family doctor. To do this, the user enters the system to request the access data. After that, the system verifies the permissions for the action to be carried out; if permitted, the synchronizer proceeds to extract the EMRs from the local database and proceeds to update them with those stored in the blockchain, as long as there is no connectivity failure. In the case of a connectivity failure, it shows the latest update of the EMRs stored in the local database. If the actor does not have the required permissions, they can make a request to the administrative authority to enable permissions.
- Case 3. Attention to the patient in a health institution. This case begins when a patient attends a medical visit to a health provider, which may be their daily provider or a new one. There, the patient is cared for by the medical staff, who, if they have the appropriate permissions, and, according to approved clinical practices, start by consulting the patient’s medical records. For this, the synchronizer extracts the existing EMRs from the local data of the hospital and then proceeds to update them with those stored in the blockchain, as long as there is connectivity. In case of a connectivity failure, the system shows the latest update of the EMRs stored in the local database. Next, the medical staff performs the patient care and generates a series of medical records that are stored by the synchronizer: first, in the local database of the health provider, and then, if there is a connection to the network, it proceeds to send the transactions to the blockchain. If there is no connection, the synchronizer marks the new records as not sent and waist until there is a connection to send them to the blockchain, thus making them available for all the nodes of the system. In case of not having the required permissions, a request is made to the patient or the family doctor for access.
- Case 4. Consultation with an external provider. This case begins when a patient attends a consultation with an external doctor or a laboratory. In this appointment, the doctor accesses the patient’s data through a web interface; however, generally, this external provider has limited access to the patient’s EMRs. At the end of the session and if the doctor has the appropriate permissions, they can enter the new records into the patient’s medical record on the blockchain. In the case of not having the required permits, a request is made to the patient or family doctor.

### 3.3. Design of the Architecture

**Figure 4:**
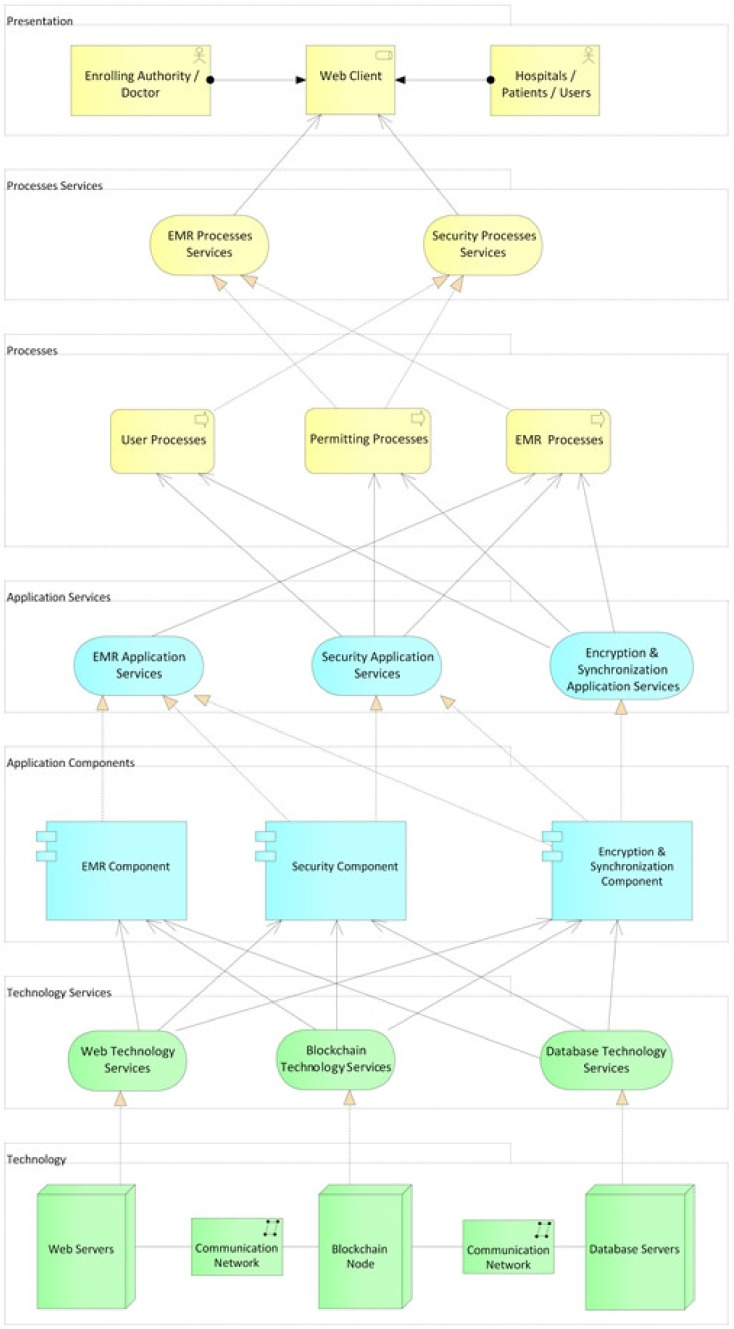
HealthyBlock architecture—layered viewpoint.

In this section, the design components and layers of the HealthyBlock architecture are presented. In order to show the relationships between layers and components, a layered viewpoint, using the ArchiMate^®^ 3.1 (The Open Group, San Francisco, CA, USA) specification, was developed, as shown in Figure 4. The design serves as a guide to develop blockchain-based, unfragmented electronic medical record systems with data integrity, usability, security, privacy, and resilience characteristics in connectivity failure scenarios. As can be seen, the HealthyBlock architecture is made up of the following layers: technology, technology services, application components, application services, processes, processes services, and presentation.

A description of the proposed layers and their components is presented below.

#### 3.3.1. Technology Layer

This layer is made up of the hardware components of the architecture, such as servers and communication equipment, which are necessary to create the network that allows the operation of the EMR system. It is also responsible for the physical storage of medical records, either on the blockchain so that they are available to all the actors in the system or in the local databases of medical service providers. Three types of servers are proposed:

- Web servers (hardware): these elements host part of the system for encryption, obfuscation, and synchronization of electronic medical records. These servers are configured with the operating system and the web server (software) chosen to host the applications.
- Local database servers (hardware): these servers are configured with the operating system and the chosen database server (software) to store the clinical information of the patients locally. Hospitals may have their own information systems, but they must provide a two-way communication channel with the HealthyBlock system, which allows obtaining local data to be published on the blockchain and made available to all actors, and receiving patient data, which should be included in the patient’s local medical history.
- Servers (hardware) to host the nodes of the blockchain network: these servers are configured with the operating system, the blockchain on which the smart contracts will run, and a blockchain client in charge of providing the configuration interface for the nodes and managing the connection with other nodes to create the blockchain network. An important element when configuring the blockchain node is the choice of the consensus algorithm, for which the proof of authority (POA) algorithm is recommended given its advantages for private blockchain networks. It is also suggested that health institutions have a blockchain server to support network maintenance and guarantee access to the most up-to-date synchronized information even under a disconnection scenario.

It is important to indicate that the institutions can choose to have a single server (hardware) on which the operating system, the web server (software), the database server (software), the blockchain, and the blockchain client are installed, but with a potential compromise on performance.

#### 3.3.2. Technology Services Layer

This layer is responsible for exposing the services offered by the technology sublayer and provides communication between the latter and the application components sublayer. The services offered by this layer are the blockchain service, web service, and database service.

The functions of the blockchain service are (i) establishing the connection to the blockchain, performing two specific functions for this (first, it acts as a wallet that allows system users to have a unique identity to control their interactions with smart contracts, and, second, it establishes a bridge for the upper layers of the architecture to connect with the blockchain network, allowing them to execute actions and receive data from the blockchain), and (ii) using the blockchain as a repository of information from medical centers such as hospitals and references of patient affiliation to different medical centers, carrying out the process of saving user data and all their medical information, such as treatments and medicines, on the blockchain.

The web service is in charge of receiving the requests made by the upper layers and managing them on the web server. This web service must be able to (i) make bidirectional and/or unidirectional and synchronous or asynchronous connections, (ii) implement a web information transfer and delivery protocol to the upper layers, in order to have access to all the functionalities proposed by the HealthyBlock architecture, (iii) encrypt or obfuscate medical history data, and (iv) synchronize clinical data from local hospital databases with clinical data stored on the blockchain. The database service controls and accesses the databases that each medical entity has to locally store the clinical history of their patients.

#### 3.3.3. Application Components Layer

This layer is made up of three major components called the EMR Component, the security component, and the encryption and synchronization component. These elements are responsible for providing the services related to electronic medical records, users, security, and privacy. The functionality of these components is detailed below.

##### EMR Component

**Figure 5:**
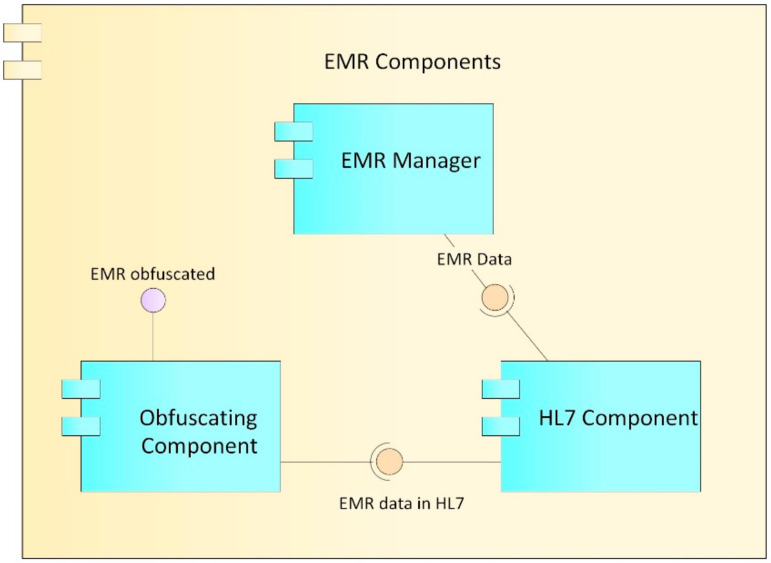
Electronic medical record (EMR) components.

The EMR component (Figure 5) is made up of the EMR manager, HL7, and obfuscation components, which together provide the functionalities for the record management.

- EMR manager: This component is in charge of managing the data structure of the records, including the different sections and fields that the records have. It works in conjunction with the synchronizer component to which it sends the captured EMR information.
- HL7 component: For the construction, storage, and transmission of the records, according to the Health Level Seven International (HL7) clinical standard. This component oversees formatting the EMR data such that they comply with the rules established in HL7.
- Obfuscation component: Obfuscation consists of replacing or altering the existing real data to hide details and protect either the identity or the sensitive characteristics of the information of a user. Ideally, the technique should resist reverse engineering attacks [59]; however, at the same time, it should not alter the data in excess, so that it remains useful for some level of decision-making. Data obfuscation techniques have three main properties: reversibility, specification, and change [60]. In accordance with these concepts, the proposed obfuscator alters the clinical data of the patients in order to be able to carry out trend analysis on them, but without being able to identify the real data or its owner. This component performs the obfuscation process on the basis of the access levels proposed in [61] and defined in the role manager component described below.

##### Security Component

**Figure 6:**
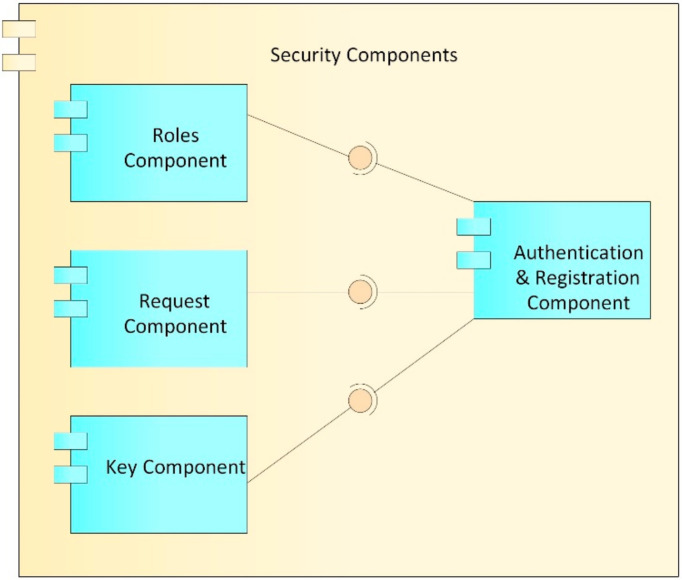
Security components.

The security component (Figure 6) is in charge of the roles of users, requests for access to the records, and the keys (public and private) of system users and, therefore, provides authentication of access to the EMRs stored in the blockchain and local databases.

**Figure 7:**
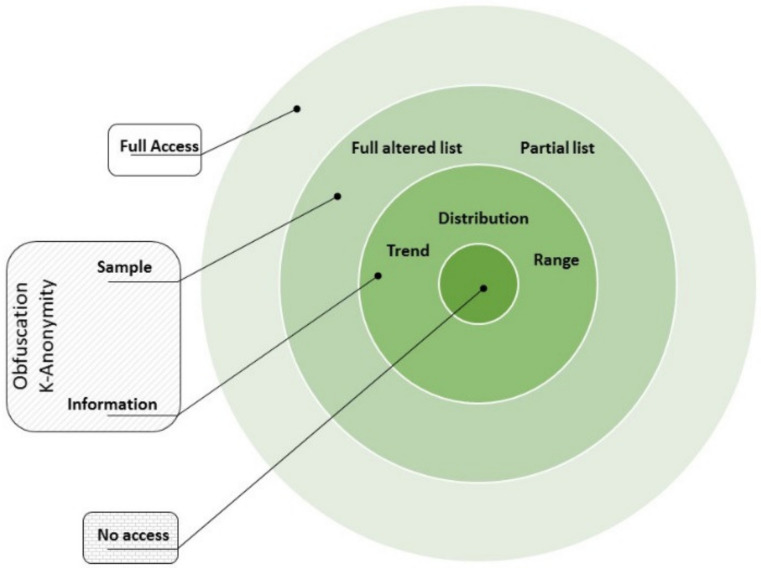
Access types.

- Roles component: This component is in charge of managing the roles assigned to each user within the system. Each user who is registered in the system is assigned a role (patient, doctor, laboratory, administrative, etc.), which in turn has a defined series of differential accesses which refer to the assignment of a set of predetermined permissions, in order to reduce the complexity of establishing a user access to clinical information for a user/patient. The proposed access levels in Figure 7 are based on those defined in [61]: (i) no access, where no information is revealed from the patient; (ii) information, where just basic descriptors of the general behavior of the data are shared but no specific data are revealed (range, trend, statistical distribution); (iii) sample, where the shared information is restricted in quantity or quality of the data (last data, every two data points, truncated or obfuscated data); (iv) full access, where the entire dataset is shared without alteration.

- Requests component: This component is in charge of managing requests for access to a patient’s clinical data. These requests can be issued by a doctor or by an entity and must be approved by the patient or the family doctor. In case they are approved, this component creates a relationship of the patient’s records with the user to whom the request was approved, as well as the type of role that is granted in the system.
- Keys component: This component is in charge of generating and managing user accounts with which you can interact and carry out transactions on the blockchain. While, in a conventional system, a user/password pair is used to authenticate, in the blockchain, accounts whose access credentials are a cryptographic pair are used, which, for security, must be made up of a public key and a private key, which can be seen as the username and password of the blockchain. As can be deduced from the above, these accounts are necessary for interaction with the blockchain; therefore, each user in the system must be associated with an account on the blockchain, with their public key and private key being the access credentials. Likewise, the keys component generates the public and private keys used by the encryption component to carry out the encryption processes; the generation, management, and correct validation of these keys are of utmost importance, in order to carry out a correct encryption of the EMR.
- Authentication and registration component: This component is in charge of registering and authenticating the different actors that have access to the system, such as the administrative authority, doctors, hospitals, clinical centers, and patients. At the time of registration, the user is assigned a name, a password, a role, and an access level that, by default, is without access. Likewise, the keys component generates the public and private key for access to the blockchain, and the public and private keys of the blockchain are stored in the user’s account of the system to facilitate later access. Later, public and private keys are generated for the encryption processes; the public key is stored in the blockchain so that it can be used by all the actors in the system who want to communicate with the user, while the private key is delivered to the user for custody. At the time of access to the system, the user uses the username, password, and private encryption key that was assigned. If the authentication process is correct, this component allows access to the system and, in collaboration with the roles component, grants the user the access levels assigned to them for CRU operations on the EMR system. This authentication component is also responsible for generating the log of the transactions carried out by users on the system, in order to carry out audit processes of visualizations, additions, or modifications to the electronic medical records (EMRs).

##### Encryption and Synchronization Component

**Figure 8:**
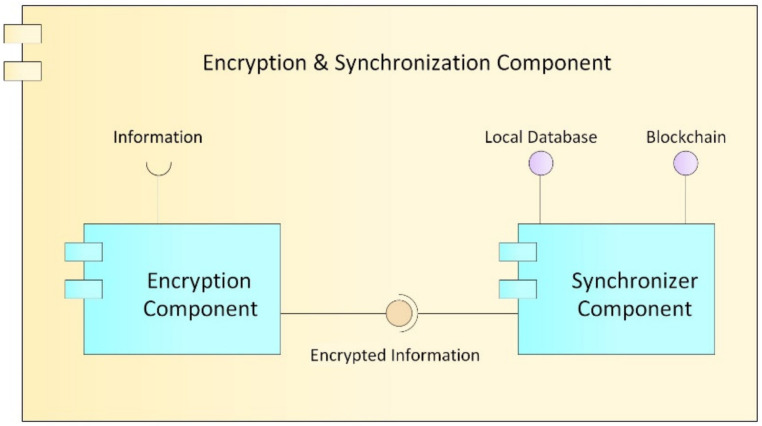
Encryption and synchronization component.

This component, as its name implies (Figure 8), provides the security and synchronization of the electronic medical records (EMRs) stored in the blockchain with those of local data from clinical providers.

**Figure 9:**
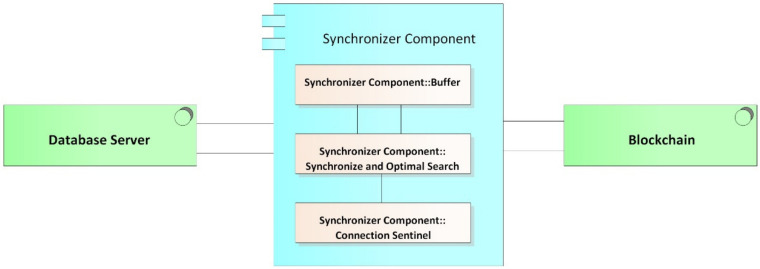
Synchronizer buffer.

- Encryption component: Cryptography is an encryption technique that disorganizes information in a particular way so that only the user can read and process it [62]; there are two types of cryptography techniques: secret key cryptography and public key cryptography. In secret key cryptography, a single key is used with which both the sender and the receiver encrypt and decrypt the data; this is also known as symmetric encryption. Public key cryptography is made up of two encryption systems that give rise to a public key that is shared with all those to whom it wants to communicate and a private key that is secret [63]. In the proposed HealthyBlock architecture, public key cryptography is suggested, since it offers greater security, and special care must be given to the delivery of the private key to system users.
- Synchronizer component: This component is in charge of creating the bridge between the blockchain network and the internal databases of the medical centers. The structure of the module can be seen it Figure 9, and the main functions of the synchronizer are as follows:
  ○ Detect system connection failures to the blockchain network, through a connection sentinel.
  ○ Store transactions (including user information, permissions, and the medical records of a patient) in the local database of the medical provider to later send them to the blockchain.
  ○ Manage the transmission of transactions to the blockchain, implementing a buffer to avoid errors in the block creation of a disconnected node. The intermediate buffer can be implemented using a queue management algorithm or other existing techniques for managing buffers available in the literature.
  ○ Download the information of users and patients from the blockchain when it is requested by medical staff and it is not stored in the local database; this means that the synchronizer works on demand, which reduces the work load of the component and reduces the amount of information stored on the local database. For example, if a patient has an appointment at a new health provider, the patient’s EMRs are downloaded from the blockchain and saved in the local database. If the hospital is experiencing a connectivity failure, the synchronizer updates the information in the local database from the latest version of the local blockchain node; thus, the system guarantees access to the latest data on the blockchain before the failure. Once the connection is re-established, even during patient care, the synchronizer notifies the doctor if there are new records on the updated blockchain.
  ○ Guarantee the resilience in the integrity of the architecture data, since, in the event of a connectivity failure of a clinical provider’s node, the synchronizer, during the disconnection period, stops sending transactions to the blockchain and only stores them in the local databases of the providers, marking them as not sent in said database. Once the synchronizer detects that the provider’s connection has returned, it proceeds to send the unsent transactions that were generated during the disconnection period, so that they can be included in the blockchain.
  ○ Execute algorithms of record searching when inserting transactions to the blockchain and when downloading information from it. It must be programmed with efficient search algorithms, in order to improve the access speeds of the required information.

#### 3.3.4. Application Services Layer

This layer is responsible for exposing the services offered by the application components layer. The services found in this layer are application services EMR, application services encryption and synchronization, and application services security. The above services are used by the process layer to carry out the functional processes of the HealthyBlock architecture and are supported by the EMR, security, and encryption and synchronization components, the operation of which was described in the previous section. It is important to indicate that the application security services is made up of the authentication, data registry, keys, and permissions services, the use of which is shown in the next section.

#### 3.3.5. Processes Layer

In this layer, three processes are executed that allow the functionality of the HealthyBlock architecture: User processes, permission processes, and EMR processes. These processes and their relationships with the components of the lower layers are described below.

**Figure 10:**
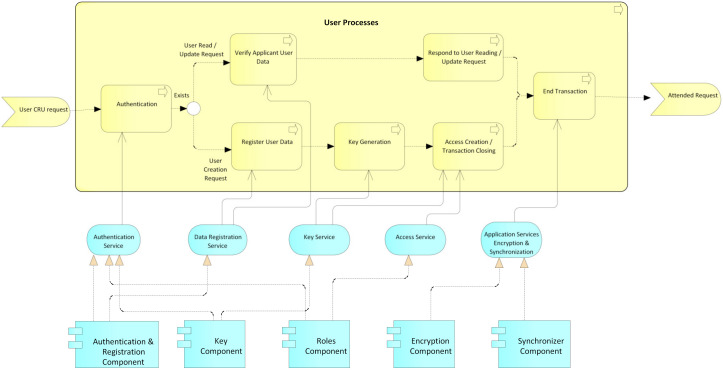
User processes.

**Figure 11:**
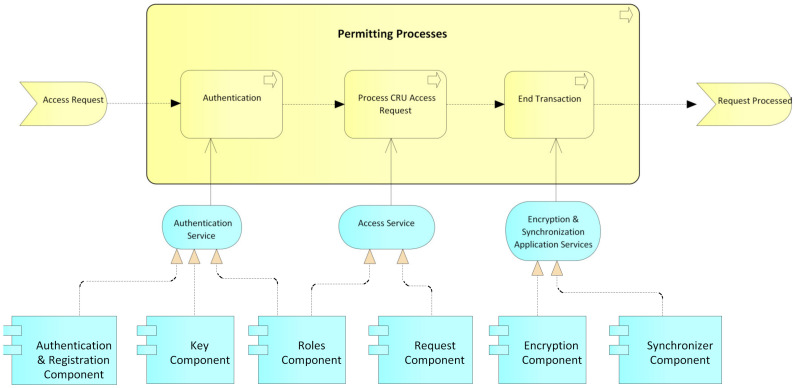
Permitting processes.

**Figure 12:**
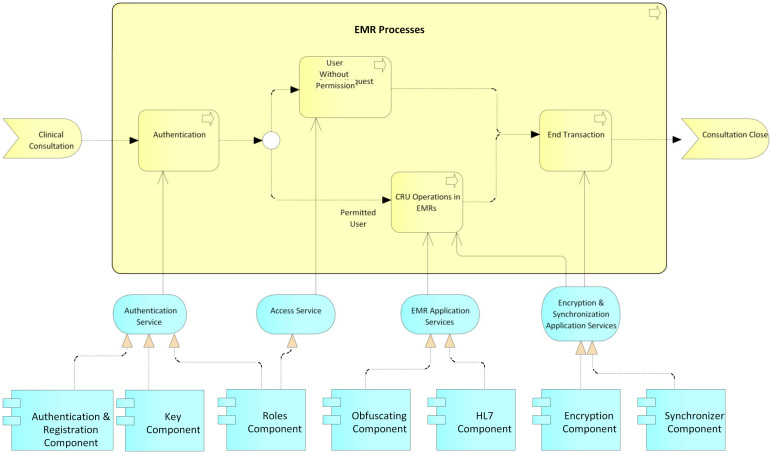
EMR processes.

- User processes: This process begins with a CRU request by an actor before an administrative authority of the system (Figure 10); then, an authentication process is performed by the administrative authority. For this, the system relies on the service authentication that is provided by the authentication, keys, and roles components. Then, it is validated whether the request is a creation or not. In the case of a creation request, the user data are registered, supported by the data registration service provided by the authentication component. The system then generates the public and private keys for the blockchain and the public and private keys for the user, while the user’s private key is delivered to them for safekeeping. Next, according to the role and type of user, the access level for the requesting user is created, supported by the access service that is provided by the roles component. Finally, the transaction is concluded, and the system, by means of the application services encryption and synchronization, which is provided by the encryption and synchronization component, takes the data of the transaction, encrypts them, and saves them on the blockchain. If it is not a creation request, but rather a read, update, or delete request, the data of the requesting user are verified, relying on the data registration service, which is provided by the authentication component, and then the request is processed. request required. Finally, the transaction and the system are concluded by means of the application services encryption and synchronization, which is provided by the encryption and synchronization component, which takes the transaction data, encrypts them, and saves them in the local database and the blockchain.
- Permits processes: This process begins with a request for permission to access a patient’s electronic medical records by an actor, generally a hospital or a doctor (Figure 11). The patient or authorized guardian then authenticates themself in the system, through the authentication service that is provided by the authentication, keys, and roles components. Next, the patient performs the CRU operation; that is, it allows access to new users, and modifies or updates access to their medical records, relying on the system access service, which is provided by the roles component. Finally, the transaction is concluded and the system, by means of the application services encryption and synchronization, takes the data of the transaction made, encrypts them, and saves them in the local database and blockchain.
- EMR processes: This process usually begins with a patient’s consultation with a medical provider (Figure 12). Next, the medical provider authenticates themself in the system through the authentication service, which is provided by the authentication, keys, and roles components. The system then verifies if the provider has sufficient permissions to view, create, or update the medical records; if so, it gives access to the EMRs and the medical provider proceeds to perform the necessary CRU operations on the patient’s EMRs, using the application services EMR, provided by the obfuscator and HL7 components, and the application services encryption and synchronization, using the encryption and synchronizer components. If the medical provider does not have access to the patient’s records, the system gives them the option to make a request to access the EMR; this access request is supported by the system access service which is provided by the roles component. In the two ways previously described, the transaction and the system are concluded by means of the application services encryption and synchronization, which takes the data of the transaction made, encrypts them, and saves them in the local database and blockchain.

#### 3.3.6. Processes Services Layer

This layer is responsible for exposing the services offered by the process layer. These services are used by the presentation layer to advance these processes. These services are divided into two groups: processes services EMR and processes services security. These services are accessed by users and base their functionality on the processes described above in the process layer.

#### 3.3.7. Presentation Layer

This is the output layer of the system. It is made up of the web client component, which, in turn, is supported by a browser through which the user can access the system. Likewise, it is made up of interfaces for clinical, patient, and medical use. These interfaces allow the consultation and management of the EMR, administrative interfaces, and local and administrative entities. The first is used by the local administrative personnel of the healthcare entities to, for example, assign a doctor to a patient. The second is used by the administrative entities to create or update the users of the system.

## 4. Developed Prototype

**Figure 13:**
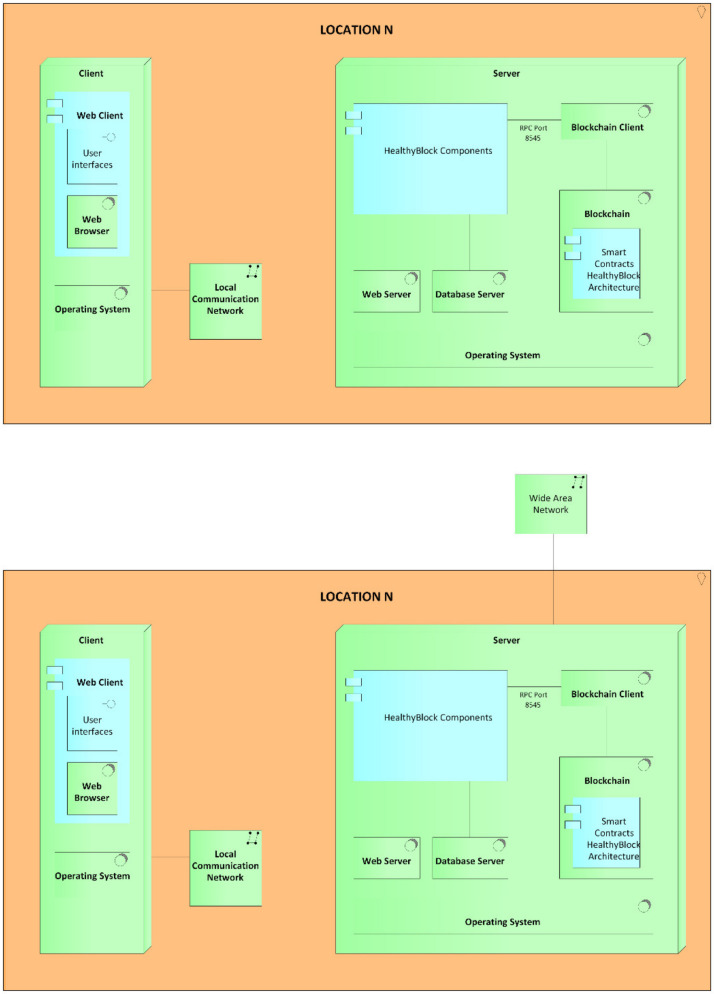
Architecture deployment diagram.

The deployment of the HealthyBlock architecture for the execution of the prototype was carried out in the test environment, detailed in the diagram shown in Figure 13. As can be seen, for the technology layer, the option of a single server (hardware) was chosen, which contained the base operating system, web server (software), database server (software), blockchain, and blockchain client. Likewise, the application components and application services layers of the HealthyBlock architecture were executed on this server, either as programmed components or as smart contracts, as explained in Appendix A. For privacy purposes, no real-life patient medical data were used in the performance tests.

In Appendix A, all the components shown in the prototype deployment model (Figure 13) are presented, as well as the development environments and contracts used to implement the components of the HealthyBlock architecture layers.

## 5. Evaluation

### 5.1. Testing Environment

For the development of the tests on the prototype, the following hardware characteristics were used in the client computers: Intel Core i3-7020U 2.30 GHz processor, 2 cores, 3 MB cache, 4 GB RAM 1200 MHz DDR4, 1 TB hard disk, and 433 Mbps 2.4 GHz/5 GHz wireless network card.

For the server computers, the hardware characteristics were as follows: Intel Core i7-870 3.6 GHz processor, 4 cores, 8 MB Cache, 8 GB 2400 MHz DDR3 RAM, 1 TB hard disk, and 1 Gbps network card. Both the client and the server used the Windows 10 (64-bit) operating system.

For the local network device, a Technicolor TC8305C Gateway was used, with broadband access via the integrated DOCSIS 3.0 (8 × 4) cable modem with 1 Gbps Ethernet ports and IEEE 802.11b/g/n 2.4 GHz wireless access points. For the wide area network, the OpenVPN application was used, which allows virtual private network (VPN) connections to be made in order to simulate geographically separated equipment.

The blockchain used for the development of the prototype given its ability to execute smart contracts was an Ethereum permissive network, which was configured with a proof of authority consensus algorithm. Smart contracts were written in Solidity. Remix IDE was used to compile and deploy the contracts on the blockchain.

**Figure 14:**
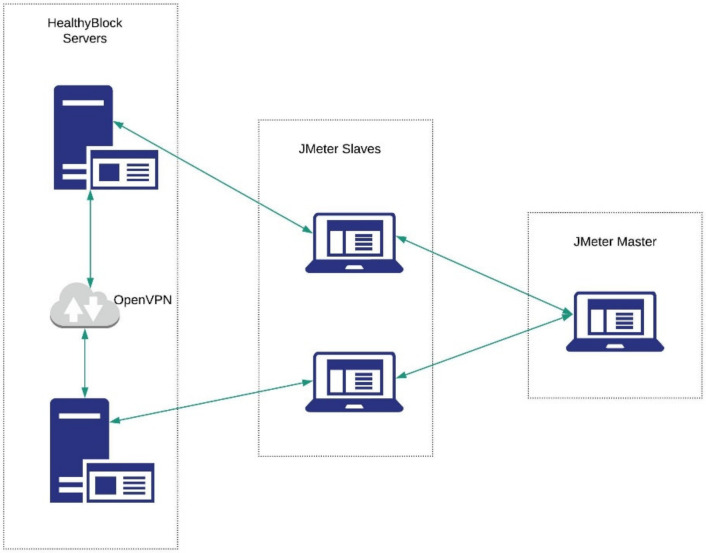
JMeter distributed configuration.

The performance of the prototype was evaluated using the System Explorer v7.0.05356 [64] (Mister Group), and Apache JMeter v5.2.1 (Apache Software Foundation, Wakefield, MA, USA) [65] tools in a distributed manner, as shown in Figure 14.

The experimental parameters are shown in Table 1.

### 5.2. Performance Indicators

The main criteria used to evaluate the performance of the prototype derived from [66,67] were as follows:

- Latency: measure of responsiveness that represents the time it takes to complete the execution of a request.
- Transaction throughput: the number of successful transactions that the prototype can process per second.
- Resource utilization: utilization of resources while processing requests and responses of transactions. Resource utilization can be measured by checking the resources used by the prototype in the CPU, memory, network, and I/O in a defined period of time.

### 5.3. Performance Results

**Figure 15:**
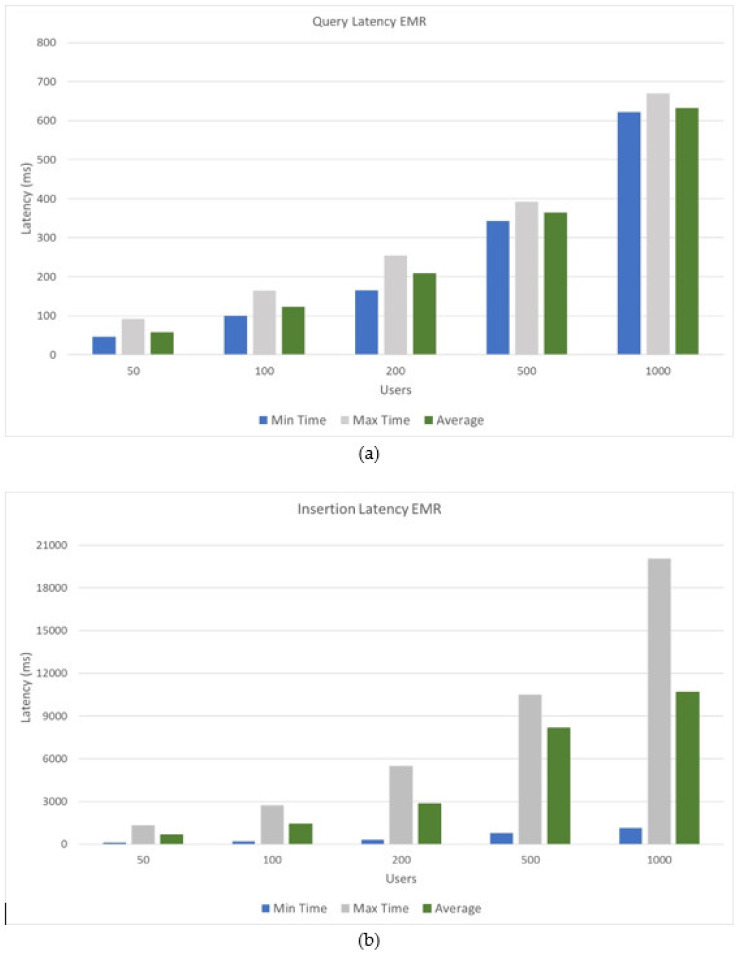
Latency results: (**a**) query latency EMR; (**b**) insertion latency EMR.

Figure 15 shows the minimum, maximum, and average latency results when executing query requests and creating electronic medical records. These response times are those used by the synchronizer to carry out all concurrent requests; however, the end user would not perceive these times, since electronic medical records are first stored in the local databases of health entities and then be uploaded to the blockchain. As can be seen, different numbers of users were used to observe the variation in the response time, compared to consultation and insertion requests. As can be seen, the query requests have lower latency since they do not need to be synchronized, obtaining for the analyzed user groups an average latency range of 58 ms to 622 ms with a linear behavior between each group of users. For requests to insert electronic medical records, response times increased, given the synchronization and validation operations that must be carried out and previously described in this document, obtaining an average latency range of 633 ms to 10,676 ms, following a linear behavior among each group of users. For the analyzed user groups (50, 100, 200, 500, and 1000), a maximum number of transactions per second was obtained for each group (110, 120, 200, 240, and 600, respectively).

**Figure 16:**
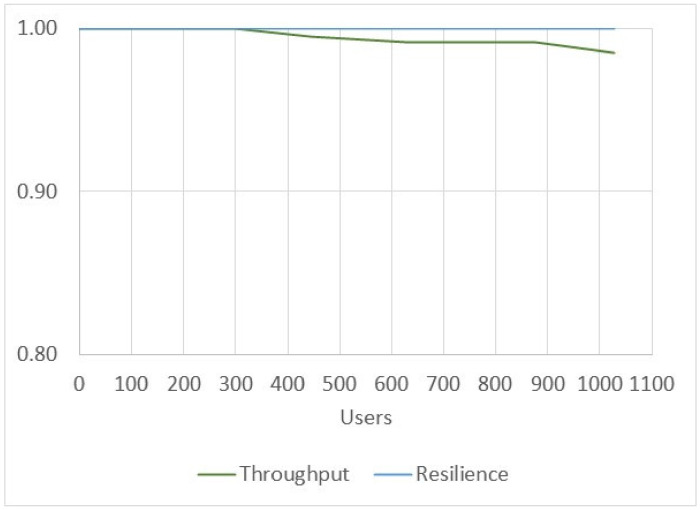
Throughput and resilience of the HealthyBlock prototype.

Figure 16 shows the performance and resilience of executing electronic medical record creation and query processes to obtain the number of successful transactions that the prototype can process and synchronize. The presented values were obtained from the transactions carried out in the previous latency tests with user groups. On average, the prototype performance was 99.54% for high-concurrency scenarios greater than 900 users and 100% for the synchronization of successful transactions made on the system.

**Figure 17:**
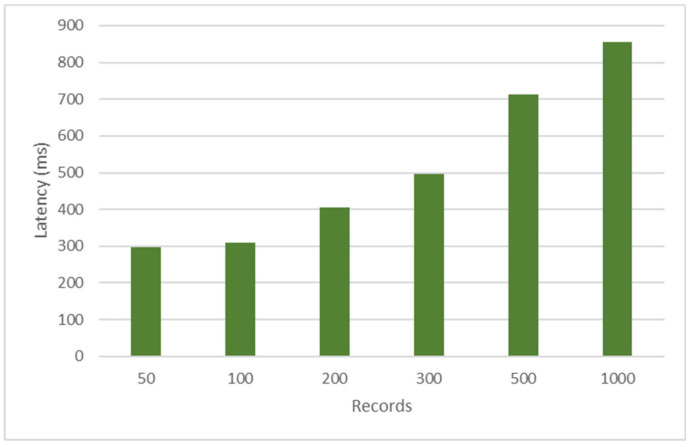
Synchronizer resilience time.

Figure 17 shows the synchronizer latency for updating a node’s local database when that node disconnects from the blockchain network and reconnects. To develop this test, which seeks to verify the resilience of the prototype in terms of response times, one of the nodes of the prototype was disconnected and 50, 100, 300, 500, and 1000 records were loaded in the node that was connected in the blockchain; then, the node that was disconnected to the blockchain was reconnected, and the update times were taken for each group of records indicated in the local database. As can be seen, the latency times had a minimum of 275 ms and a maximum of 802 ms, being optimal synchronization times for high-demand processes when the EMR information is required to be synchronized.

**Figure 18:**
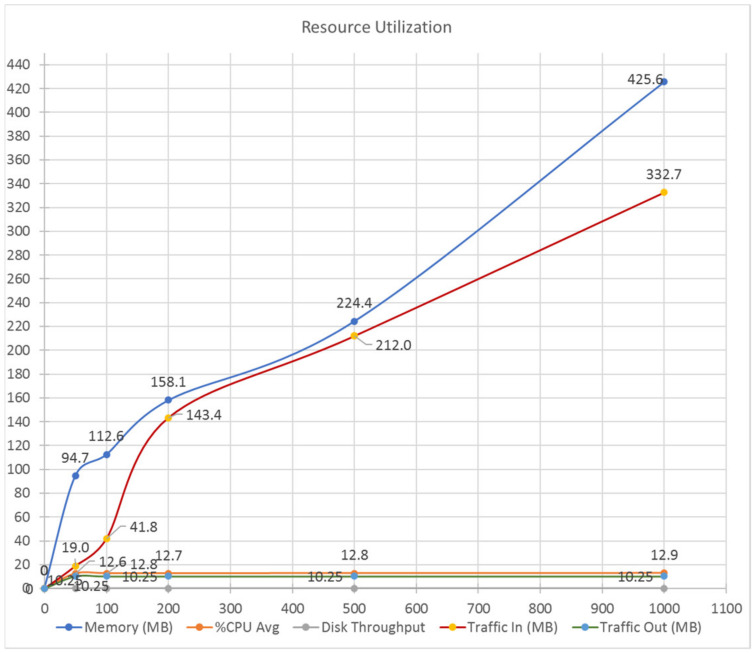
Resource utilization of the HealthyBlock prototype.

Figure 18 shows resource utilization during testing of the HealthyBlock prototype. As can be seen, system parameters such as RAM memory usage (MB), average CPU usage (%), disk performance, and incoming and outgoing traffic were measured. The graph shows that, despite increasing incoming traffic from 19 MB to 332 MB, the average CPU usage remained practically constant at approximately 13%. Memory usage for these request ranges increased from approximately 95 MB to 430 MB, representing a usage range of 1.2% to 5.3% of total available system memory; this does not represent stability problems for a server with 8 GB of memory onward running the prototype. Finally, values such as disk throughput (0.1 MB) and outgoing traffic (10.25 MB) remained constant throughout the tests carried out.

### 5.4. Functionality and Usability Evaluation

**Figure 19:**
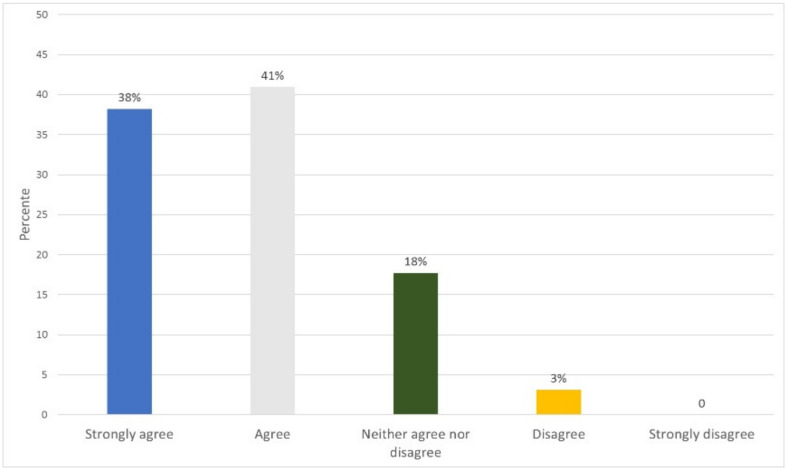
Usability survey results.

**Figure 20:**
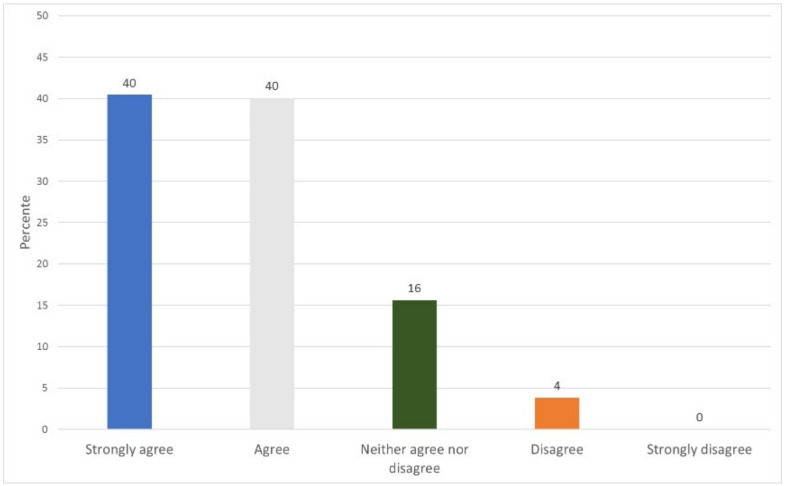
Functionality survey results.

To evaluate the usability and functionality of the prototype, a survey was conducted of 32 people related to the medical area, who performed the roles of patient, doctor, laboratory, administrator, or family doctor/staff. They answered nine questions corresponding to usability for a total of 288 answers. Eighteen questions were answered for functionality, for a total of 576 answers. Appendix B shows the instrument and how to carry out the survey. The results obtained show an acceptance of 79% of the prototype regarding its usability (Figure 19) and an 80% acceptance regarding its functionality (Figure 20).

## 6. Discussion

The proposed architecture provides a secure and resilient infrastructure for electronic medical records, which maintains an integrated view of the medical records of patients in a hospital system. Security and privacy are provided through encryption and authentication techniques across the blockchain. Likewise, the architecture proposes a private network configured with a consensus algorithm (POA), which, in addition to making the privacy of the system more robust, allows scalability, since a large number of transactions can be executed optimally given that the consensus algorithm (POA) does not require as much processing time. The use of smart contracts for access control and role definition guarantee security as to who can enter the system. An additional feature that is achieved when using blockchain networks is the immutability of EMRs, which is given by the data structure that the blockchain possesses and which guarantees the detection of subsequent changes in data and multiple copies in the network. Likewise, the consensus algorithm (POA) proposed in the architecture guarantees the veracity of the copies of the medical records at each node.

It is important to note that the storage space is going to grow rapidly, since it holds the records of all the patients in a hospital network instead of just references to their local databases, like other existing solutions. However, the advantages in terms of security and accessibility outweigh the storage costs. In addition, there is the possibility that thin clients can connect to the blockchain, without necessarily having to store the massive data structure, thus allowing the participation of a large number of users, registered on the blockchain, but without the limitation of storage, which only use the authentication and access services of the EMR system.

A key element of the HealthyBlock architecture that must be carefully programmed is the synchronizer, since it is the core of the system, due to its functions of maintaining the integrity of the system data. This element has a great impact on the latency times of the system, as observed in the performance tests carried out. During the development of the prototype, slight changes in the querying design, both on the local database and on the blockchain, generated changes in performance. The local database still needs to improve performance on EMR querying, whereby improvements can be implemented until the blockchain technology optimizes their data structures for querying.

## 7. Conclusions

In this article, HealthyBlock was proposed as an architecture based on blockchain networks, which allows the development of unified electronic medical record systems between different health providers, with resilience in the integrity of data in the event of connectivity failures and with characteristics of usability, security, and privacy, For this, the architecture proposes the use of software components that allow the detection of failures and the correct synchronization of the data between the local databases of hospitals or clinical centers and the blockchain, thus guaranteeing that the actors have secure access to consistent clinical information. Using the HealthyBlock architecture, a prototype was implemented for the care of patients in a network of hospitals, from which it was possible to observe, in the results of the evaluation, a high efficiency to keep the EMRs of patients, regardless of the clinical network provider they see, as well as residence after a connectivity failure, with very short synchronization times. The experiment demonstrates the feasibility of implementing prototypes developed from the HealthyBlock architecture in a productive environment. The implementation of the test architecture can be accessed in a GitHub repository for further study [68].

As future work, expansion of the architecture components is proposed to achieve the following:

- Patients should be able to download their records on portable media, such as cryptographically signed flash cards or cell phone APIs, in such a way that patients, when making a visit to a clinical provider, and in case the system of this provider is faced with a connection failure, can perform a wired or wireless synchronization between the most up-to-date version of the EMRs that they have and the provider’s local databases.
- Integrate clinical decision support services (CDSS) to assist physicians in making decisions about the diagnosis of their patients.
- Support patients in their health routines such as medicine schedules, medical appointment reminders, etc.

In addition, the creation of a new data structure for the blockchain was proposed, which allows not only data security but also a more efficient query, since it currently behaves as a common linked list with a large size expected to arrive. Having the linear search before large volumes of data and concurrency can generate high latency. It is proposed to continue investigating other consensus algorithms that also allow greater agility in the verification processes of blocks, even supported by thin clients that do not necessarily store the blockchain but have available computing capacity. In relation to data privacy and access control, the validation of credentials with biometrics through a mobile device is considered as a future improvement, so that the process of assigning permits can be carried out more quickly, especially when the patient is in front of the clinical actor who requires it. Finally, the definition of a more flexible medical record storage structure is essential to store a wider variety of data that a real EMR application will require.

## Figures and Tables

**Table 1:** Experimental parameters.

Variable	Levels
Number of threads (users)	50, 100, 200, 500, 1000
Ramp-up period (seconds)	60
Same user on each iteration	Yes
Duration (seconds)	300

**Figure A1:**
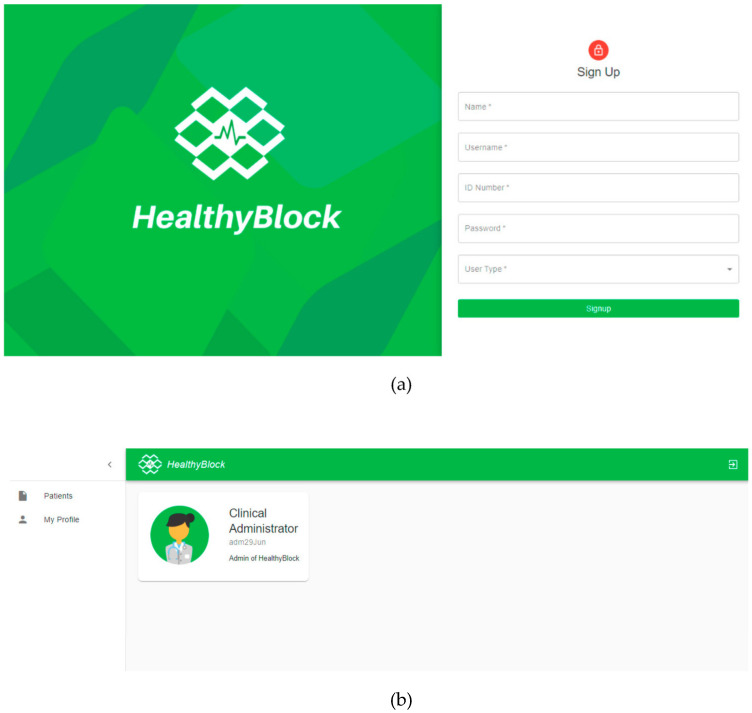
Prototype user processes: (**a**) access interface; (**b**) manager interface.

**Figure A2:**
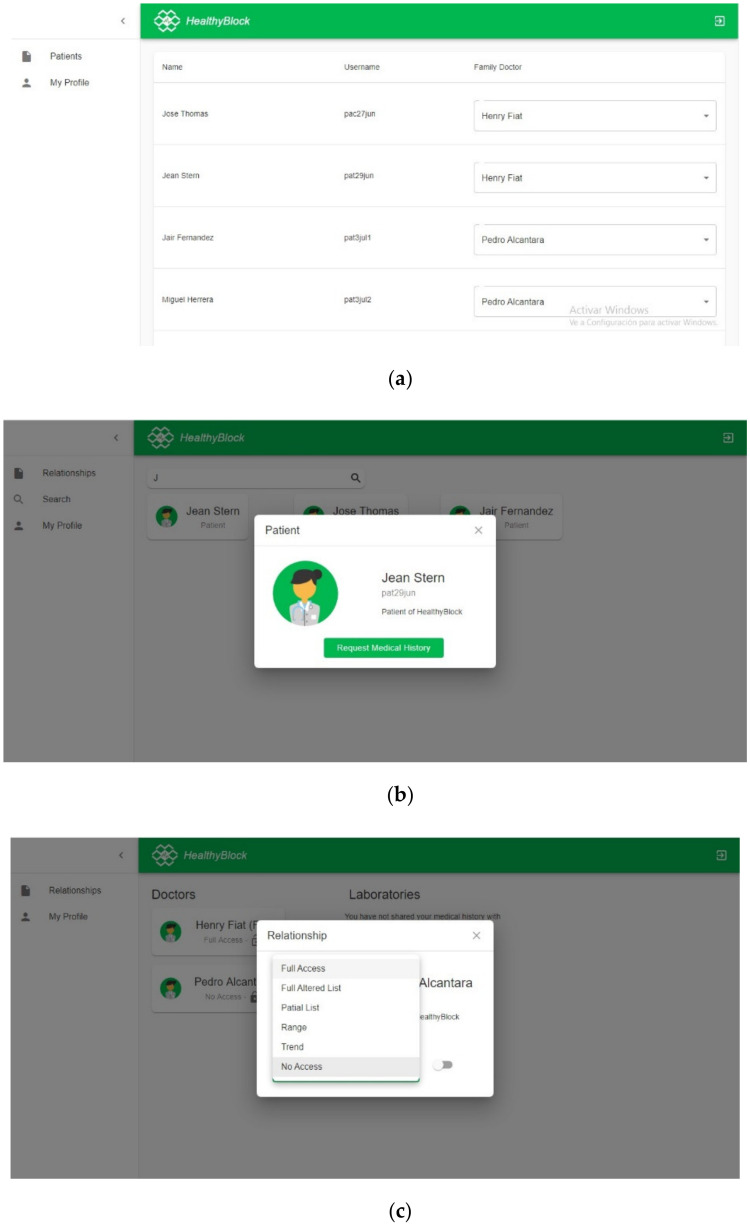
Prototype permit processes: (**a**) Manager associates family doctor with a patient; (**b**) Doctors can request access; (**c**) Patient can grant access to a medical actor.

**Figure A3:**
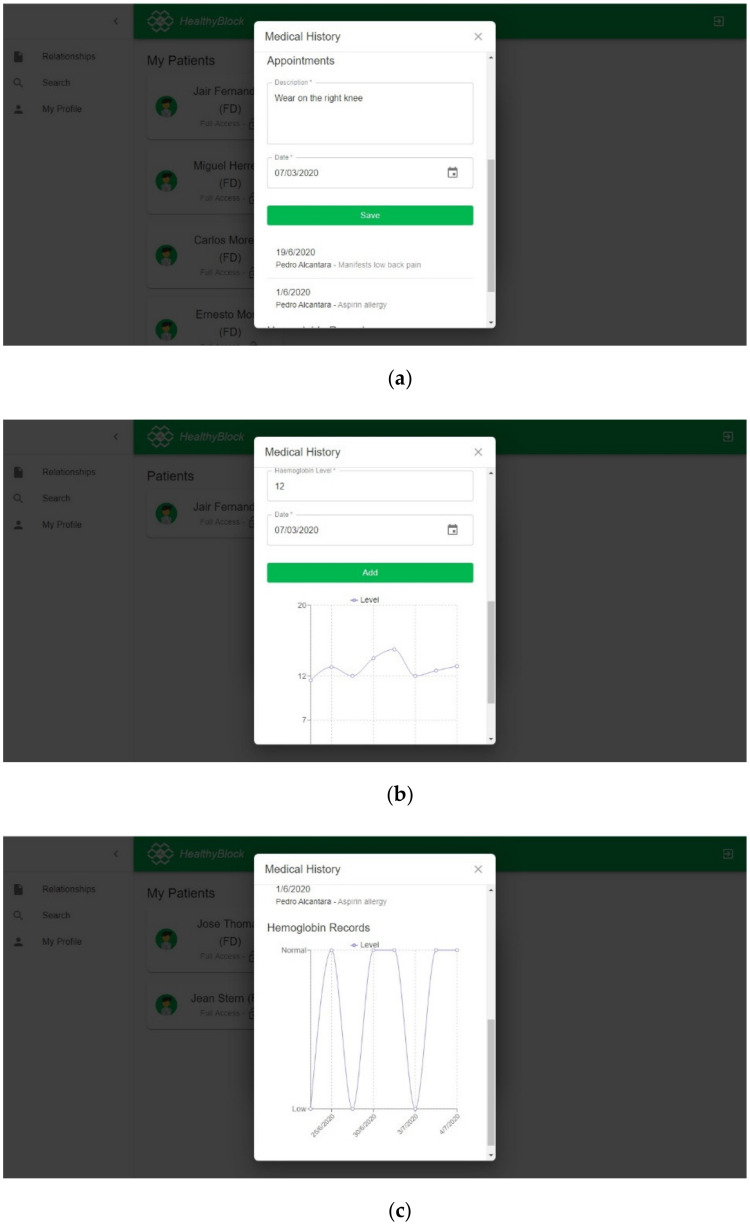
Prototype EMR processes: (**a**) Visualization of medical records; (**b**) Input of hemoglobin data of a patient by a laboratory; (**c**) Visualization of obfuscated records.

## Appendix A. Description of the Prototype

Below is a detailed description of the implementation of the prototype, used to perform the experiments and performance evaluation.

### Appendix A.1. Client

The hardware features used in the client computers were as follows: Intel Core i3-7020U 2.30 GHz processor, 2 cores, 3 MB cache, 4 GB 1200 MHz DDR4 RAM, 1 TB hard drive, and 433 Mbps 2.4 GHz wireless network card/5 GHz. The components and software used in the clients to execute the prototype are detailed below.

#### Appendix A.1.1. Operating System

The operating system used in the clients was Windows 10 (64-bit) [69].

#### Appendix A.1.2. Web Client

As indicated in the description of the architecture, this component is made up of the different interfaces (medical, patient, local administrative, and enrollment institution) offered by the system to carry out the processes (users, EMR, permits) and by a browser through from which the user can access said interfaces. For the specific case of the prototype developed, the Google Chrome web browser (Google, Mountain View, CA, USA) was used [70]. The interfaces were developed using React.js v16.12.0 [32], with the design of Material UI React v4.9.3.

### Appendix A.2. Server

The hardware features used in the server device were as follows: Intel Core i7-870 3.6 GHz processor, 4 cores, 8 MB cache, 8 GB 2400 MHz DDR3 RAM, 1 TB hard drive, and 1 Gbps network card. The components and software used on the servers to run the prototype made are detailed below.

#### Appendix A.2.1. Operating System

The operating system used in the servers was Windows 10 (64-bit) [69].

#### Appendix A.2.2. Blockchain Client

Geth 1.9.14 for Windows [71] (Ethereum Foundation) was used as a blockchain client. This client, developed in the Go language, is the command line interface to run a complete Ethereum node. Geth allows each node in the network to enable a port with the RPC (request procedure call) protocol [72], through which the transactions can be sent to the blockchain.

#### Appendix A.2.3. Blockchain

The blockchain used for the development of the prototype, given its extensive technical information, stability, and ability to execute smart contracts, was a permissioned network of Ethereum [31], which was configured with a consensus algorithm from proof of authority [43].

#### Appendix A.2.4. Database Server

PostgreSQL v12.3 [33] (PostgreSQL Global Development Group) was selected due to its good query performance thanks to the indexing options (b-tree, multicolumn, expressions, partial), parallelization of read queries and building b-tree indexes, and multi-version concurrency control.

#### Appendix A.2.5. Web Server

This component was performed using the internal server provided by ReactJS [32] (Facebook, Menlo Park, CA, USA).

#### Appendix A.2.6. HealthyBlock Architecture Components

The HL7, encryptor, synchronizer, and keys components were developed using Node.js v12.18.0 LTS, with the Express module v4.17.1, and the web3.js v1.2.4 library was used to connect the applications to the blockchain. The obfuscator and request components were developed with React.js v16.12.0 [32]. For the encryption component, the advanced encryption standard (AES) and (Rivest–Shamir–Adleman) RSA encryption algorithms [73] were used, in order to guarantee the security of the EMRs. For the HL7 component, an ORU-R01 message was used (Table A1), as it is useful for sending information about the hemoglobin records and medical appointments/visits that were the subject of this prototype and that are detailed in the functional description. In the case of the synchronizer, a Representational State Transfer (REST) API is additionally used that allows the management of users and their records.

**Table A1:** Message HL7.

Structure ORU-R01
MSH Message Header (General information about the message)
PID Patient Identification (Patient information)
[
OBR Observation Requests (Type of request observation)
{
OBX Observation Results (Results of the requested observation)
}
]
[
{
PV1 Patient Visit (Information about the patient’s visit)
NTE Notes and Comments (General information about the visit)
}
]

The EMR manager, roles, and authentication and registration components were developed using smart contracts in Solidity v0.5.0. Remix IDE V 0.6.6 was used to compile and deploy the contracts on the blockchain. These contracts are described below, the codes of which can be consulted in the project repository at [68].

- EMR manager: The smart contract that develops this component is called MedicalHistory.sol. This contract is in charge of managing the fields that have the EMRs that, for the developed prototype, were name, address, telephone number, the patient’s hemoglobin records, and patient queries.
- Roles: The smart contract developed by this component is called Agent.sol. This contract allows the definition of roles and, therefore, the different levels of access of an actor to the records of a patient. As previously indicated, the accesses are based on [61] and, in order to manage better functionality in the prototype, the following access levels were implemented: no access, range, trend, partial list, full altered list, and full access. Patients are a special agent as they are the owners of their medical history and, thus, must have this additional attribute; therefore, an additional smart contract was used that extends from Agent.sol.
- Authentication and registration: The smart contract developed by this component is called Platform.sol. This contract has the agents or actors of the system such as patients, doctors, laboratories, and the relationships between them; this is why this contract is considered of utmost importance, since it allows a relationship between the different system contracts.

### Appendix A.3. Network Devices

#### Appendix A.3.1. LAN Devices

A Technicolor TC8305C gateway was used to create the local area network, with broadband access via the integrated DOCSIS 3.0 (8 × 4) cable modem with 1 Gbps Ethernet ports and IEEE 802.11b/g/n 2.4 GHz wireless access points.

#### Appendix A.3.2. WAN Devices

For the implementation of the wide area network, the OpenVPN application [74] was used, which allows VPN connections to be made to simulate geographically separated equipment.

### Appendix A.4. Description of the Prototype

The functionality of the prototype is described below on the basis of the processes contained in the processes layer of the HealthyBlock architecture.

#### Appendix A.4.1. User Processes

The user CRU processes described in Section 3.3.5 are carried out in the system through the screens shown in Figure A1; in (a), the new users are registered and, in (b), having the administrator role, they can be read, updated, or deleted.

As previously mentioned, at the level of security and encryption in the prototype, the RSA and AES encryption algorithms were implemented. When a new user is registered, the key service, provided by the key component, generates a private and a public key using the RSA algorithm. The former is delivered to the user and the latter is stored in the blockchain through the agent contract. If the registered user is a patient, the system also asks them to create a password, which is later used by the encryption service to encrypt the medical records using AES.

#### Appendix A.4.2. Permission Process

The permission CRU processes described in Section 3.3.5 are performed in the system through the screens shown in Figure A2; in (a), initially, the system administrator associates patients with a family doctor, who by default has full access to electronic medical records (EMRs); in (b), through the search option, doctors or laboratories can search for patients to request access to their electronic medical records (EMR); in (c), upon entering the system, a patient can know which actors have access to their data and can also process the access requests they have made. When a patient gives a medical actor access to their EMRs, the encryption service provided by the encryption component takes the public key of the applicant and, together with the password previously created by the patient, generates a unique encrypted key through RSA encryption, which is stored on the blockchain through the platform contract. There, a relationship is established between the patient and the owner of the public key. This encrypted key is unique for each relationship that is established and is used by the owner of the public key to decrypt the patient’s EMRs in conjunction with their private key.

#### Appendix A.4.3. EMR Processes

The EMR CRU processes described in Section 3.3.5 are performed in the system through the screens shown in Figure A3; in (a), upon entering the system, a medical user is able to view and enter records of a patient; in (b), a laboratory that has a full access level may enter hemoglobin readings and the date of that reading; in (c), the display of the historical records of hemoglobin are obfuscated according to the type of access that the actor has.

When a clinical actor wants to access the EMRs of a patient, the authentication service and the access service verify that the user has the permissions to access them. In case the actor can, the key service extracts the encrypted key from the blockchain. This key is taken by the encryption service provided by the encryption component, which, together with the user’s private key, proceeds initially through RSA cryptography and later through AES cryptography to decrypt the EMRs. Then, the synchronization service, provided by the synchronizer component, extracts the data of the blockchain, in HL7 format. The opposite case occurs when an EMR must be encrypted to be stored on the blockchain; for this, the EMR service processes the records in HL7 format so that the encryption service, provided by the encryption component, uses the AES algorithm to code the data. Once the data are encrypted, the synchronization service proceeds to save them in the blockchain.

## Appendix B. Functionality and Usability Survey

To carry out the usability and functionality tests, two surveys were designed, which were answered by users related to the clinical setting, to evaluate the platform. For this, a series of questions were presented to the user who expressed their level of satisfaction with the following scale: “strongly agree”, “agree”, “neither agree nor disagree”, “disagree”, and “strongly disagree”.

Each participant registered and assumed a role in the system. The actions carried out by each of the roles on the system are described below.

- Patient: the surveyed individual answered requests from laboratory and medical users for access to their medical history and assigned a level of access to each one if they accepted the request.
- Doctor: in the search module, the surveyed individual searched for a patient and asked them for access to the medical history. Depending on the level of access, if this user could modify the patient’s medical history, they proceeded with the respective test.
- Laboratory: the surveyed individual carried out the search for patients and requested their medical history. Once the request was answered and an access level was assigned, the surveyed individual added data to the patient’s medical record.
- Administrator: the surveyed individual was in charge of assigning a patient to a family/personal doctor, who could be any medical-type user registered in the system.
- Family/personal physician: the surveyed individual had the same functionalities as a normal physician, but also could manage the access permissions to medical records of their patients assigned by an administrator.

The activities performed by the surveyed individuals are shown in Table A2.

**Table A2:** Activities performed by surveyed individuals.

Activity	Duration	Type of Interaction
Interaction with web platform	20 min	Use the web platform, assuming a certain role: doctor, laboratory, patient
Usability survey	5 min	Answer the survey
Functionality survey	5 min	Answer the survey

The questions and results obtained in the survey are shown in Table A3 for usability and Table A4 for functionality.

**Table A3:** Usability survey and answers.

No.	Questions	Strongly Agree	Agree	Neither Agree nor Disagree	Disagree	Strongly Disagree
1	It was easy to learn how to use the system.	10	10	7	5	0
2	The information provided by the system is easy to understand.	12	14	6	0	0
3	The system has all the functionalities and tools that you expected it to have.	12	15	5	0	0
4	The organization of the information on the screen is clear.	13	14	5	0	0
5	Overall, I am satisfied with how easy the system was to use.	10	11	7	4	0
6	The system interface is nice.	13	13	6	0	0
7	I like to use the system interface.	14	13	5	0	0
8	I can effectively perform operations using the system.	13	14	5	0	0
9	Finding information or functionality of the system was easy.	13	14	5	0	0
	Total (*N*)	110	118	51	9	0
	Percentage (%)	38	41	18	3	0

**Table A4:** Functionality survey and answers.

No.	Questions	Strongly Agree	Agree	Neither Agree nor Disagree	Disagree	Strongly Disagree
1	User registration is easy to perform and runs smoothly.	10	9	9	4	0
2	It is easy to log in to the website.	6	10	7	9	0
3	The user can see the access control relationships they are in (users with access to the medical record) and these are easily accessible in the interface.	16	12	4	0	0
4	The patient can modify permissions to decide who can see and/or modify their medical history in a satisfactory way.	16	11	5	0	0
5	The patient can activate the access requests of other users to their medical history (set status on).	16	12	4	0	0
6	The patient can view their medical history without problems.	14	14	4	0	0
7	The process of using the tool to interact with the system is easy and understandable.	10	6	7	9	0
8	The doctor can perform successful patient searches on the system.	15	13	4	0	0
9	The doctor can request a medical history from the patient.	14	13	5	0	0
10	The doctor can see the list of patient medical records to which they have access.	11	16	5	0	0
11	The doctor can see the medical records of the patients according to the level of access they have.	16	12	4	0	0
12	The family doctor can see and/or modify the medical history of their patients.	12	15	5	0	0
13	The family doctor can manage the medical history of their patients. (change access, change status)	12	14	6	0	0
14	The laboratory can perform satisfactory patient searches on the system.	12	16	4	0	0
15	The laboratory can make a request for clinical history to the patient.	14	14	4	0	0
16	The laboratory can view the list of patient medical records to which it has access.	13	15	4	0	0
17	The laboratory with full access can add records to the clinical history of its patients.	13	14	5	0	0
18	The laboratory can view patient records on the basis of the level of access it has.	13	15	4	0	0
	Total (*N*)	233	231	90	22	0
	Percentage (%)	40	40	16	4	0
